# Hepatic Fasting-Induced PPARα Activity Does Not Depend on Essential Fatty Acids

**DOI:** 10.3390/ijms17101624

**Published:** 2016-09-24

**Authors:** Arnaud Polizzi, Edwin Fouché, Simon Ducheix, Frédéric Lasserre, Alice P. Marmugi, Laila Mselli-Lakhal, Nicolas Loiseau, Walter Wahli, Hervé Guillou, Alexandra Montagner

**Affiliations:** 1 INRA ToxAlim, 180, Chemin de Tournefeuille, 31027 Toulouse Cedex 3, France; Arnaud.Polizzi@toulouse.inra.fr (A.P.); Edwin.Fouche@toulouse.inra.fr (E.F.); simon.ducheix@yahoo.fr (S.D.); Frederic.Lasserre@toulouse.inra.fr (F.L.); alice.p.marmugi@gmail.com (A.P.M.); Laila.Lakhal@toulouse.inra.fr (L.M.-L.); Nicolas.Loiseau@toulouse.inra.fr (N.L.); Walter.Wahli@ntu.edu.sg (W.W.); 2 Lee Kong Chian School of Medicine, Nanyang Technological University, 637553 Singapore, Singapore; 3 Center for Integrative Genomics, University of Lausanne, 1015 Lausanne, Switzerland

**Keywords:** nuclear receptors, PPARα, dietary fatty acids, fasting, steatosis, polyunsaturated fatty acids

## Abstract

The liver plays a central role in the regulation of fatty acid metabolism, which is highly sensitive to transcriptional responses to nutrients and hormones. Transcription factors involved in this process include nuclear hormone receptors. One such receptor, PPARα, which is highly expressed in the liver and activated by a variety of fatty acids, is a critical regulator of hepatic fatty acid catabolism during fasting. The present study compared the influence of dietary fatty acids and fasting on hepatic PPARα-dependent responses. *Pparα^−/−^* male mice and their wild-type controls were fed diets containing different fatty acids for 10 weeks prior to being subjected to fasting or normal feeding. In line with the role of PPARα in sensing dietary fatty acids, changes in chronic dietary fat consumption influenced liver damage during fasting. The changes were particularly marked in mice fed diets lacking essential fatty acids. However, fasting, rather than specific dietary fatty acids, induced acute PPARα activity in the liver. Taken together, the data imply that the potent signalling involved in triggering PPARα activity during fasting does not rely on essential fatty acid-derived ligand.

## 1. Introduction

In mammals, the liver plays a critical role in controlling fatty acid homeostasis, which is under tight transcriptional control. Genes involved in fatty acid biosynthesis are induced in response to feeding [1]. This anabolic response, i.e., de novo lipogenesis, is largely under the control of ChREBP [2] and SREBP1c [3], which are glucose- and insulin-sensitive transcription factors, respectively. In response to fasting, a broad catabolic response occurs in hepatocytes using free fatty acids released from adipocytes and involving several transcriptional regulators in the liver [4]. Among these molecules, Peroxisome Proliferator-Activated Receptor alpha (PPARα) is a major player, as deletion of the gene in mice leads to steatosis, hypoglycaemia, hypothermia, and reduced ketone bodies in response to fasting [5,6].

Like other PPAR isotypes (β/δ and γ), PPARα is a member of the nuclear hormone receptor family. The PPARs regulate gene expression as heterodimers with the Retinoid X Receptors (RXRs), binding to response elements in the regulatory regions of target genes. Fatty acids and their derivatives act as ligands that activate PPARα by promoting the recruitment of co-activator proteins, such as CBP/p300 and SRC/p160 [7,8]. Many different lipids, including polyunsaturated fatty acids (PUFAs) [9,10], lipoxines [9,11], and phospholipids [12,13], have been described as influencing PPARα activity. We recently reported that hepatocyte PPARα acts as a transcriptional sensor for free fatty acids released from adipocytes during fasting and controls the expression of hundreds of genes involved in fatty acid uptake, transport, and catabolism in hepatocytes [14].

We questioned whether dietary fatty acids, which induce remodelling of lipid tissue composition, may influence the fasting-induced PPARα-dependent response. To challenge this hypothesis, we designed several diets. The reference (REF) diet provided balanced fatty acid intake (n-9, n-6, and n-3 fatty acids), whereas the FISH diet was supplemented with long-chain essential fatty acids from the n-3 series, which are known to influence hepatic gene expression. In addition, a diet was designed that was deficient in essential fatty acids (EFAD) and another that was fat-free (FF). We focused on gene expression in the liver and showed that the response to fasting is triggered regardless of whether the diet mice were previously fed. The data suggest that a broad range of lipid species that do not rely on specific fatty acid series included in the diet can activate PPARα and that key signalling pathways, such as those sensing hormonal changes induced by fasting, control hepatic PPARα activity.

## 2. Results

### 2.1. Effect of Dietary Fatty Acids on Body Weight and Hepatic Fatty Acid Composition

**Figure 1:**
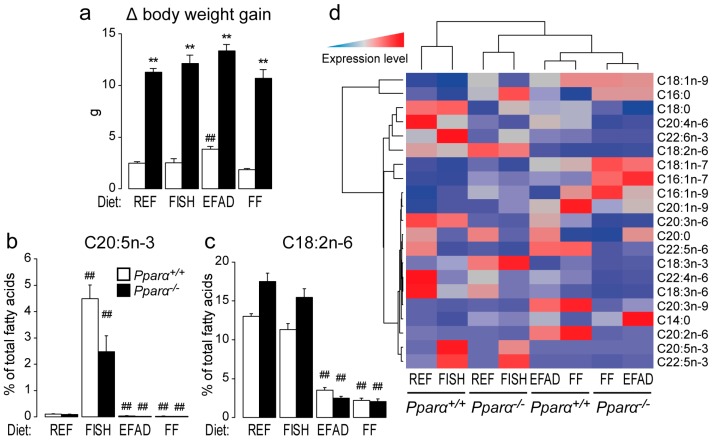
Effect of dietary fat on hepatic lipid composition. Wild-type (*Pparα^+/+^*) and total *Pparα* knockout (*Pparα^−/−^*) mice fed a standard diet (REF), a diet enriched in n-3 essential fatty acids from fish oil (FISH), an essential fatty acid-deficient (EFAD) diet, or a fat-free (FF) diet ad libitum for 10 weeks were euthanized at ZT14 in the fed state. (**a**) Determination of the delta body weight gain; (**b**,**c**) quantification of hepatic C20:5n-3 eicosapentaenoic acid (**b**); C18:2n-6 linoleic acid (**c**); and (**d**) hierarchical clustering of major hepatic fatty acids quantified by gas chromatography. *n* = 6–8 mice/genotype/diet. Data represent mean ± SEM. * significant genotype effect, ^#^ significant effect of diet composition. ** or ^##^ *p* ≤ 0.001. REF: standard diet; FISH: diet enriched in n-3 essential fatty acids from fish oil; EFAD: essential fatty acid-deficient diet; FF: fat-free diet.

First, we found that body weight gain was higher in *Pparα^−/−^* mice than in wild-type mice, independent of the diet (Figure 1a). To assess the effect of diet on liver fatty acid composition, the total fatty acid pattern was established in fed and fasted mice of both genotypes (Table S1). As expected, the FISH diet increased hepatic C20:5n-3 compared to the REF diet in both wild-type and *Pparα^−/−^* mice (Figure 1b). In addition, the EFAD and FF diets reduced the abundance of hepatic C18:2n-6 compared to the REF diet in both wild-type and *Pparα^−/−^* mice (Figure 1c). Importantly, whatever the diet, the absence of PPARα led to a major shift in the hepatic fatty acid profile (Figure 1d), providing further in vivo evidence that this nuclear receptor is a major regulator of hepatic fatty acid homeostasis in response to changes in fatty acid intake.

### 2.2. Effect of Dietary Fatty Acids on Plasma Biochemistry

**Figure 2:**
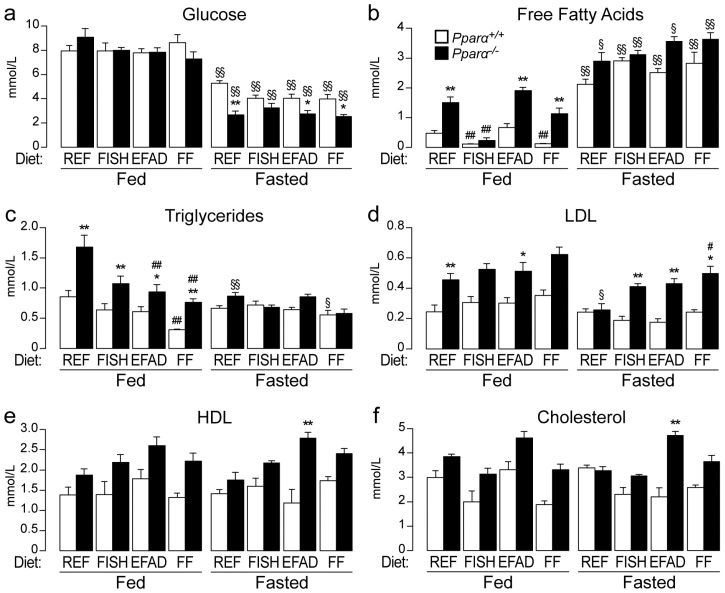

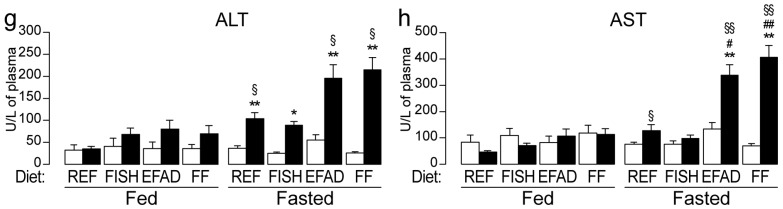
Effect of dietary fat on plasma biochemistry. Wild-type (*Pparα^+/+^*) and total *Pparα* knockout (*Pparα^−/−^*) mice were fed ad libitum or fasted for 24 h and then euthanized at ZT14. Quantification of plasma glucose (**a**); free fatty acids (**b**); plasma triglycerides (**c**); LDL cholesterol (**d**); HDL cholesterol (**e**); total cholesterol (**f**); alanine transaminase (ALT) (**g**); and aspartate transaminase AST (**h**) activity is given as mean ± SEM. *n* = 6–8 mice/genotype/diet. * Significant genotype effect, ^#^ significant effect of diet composition, ^§^ significant effect of fasting. *, ^#^, ^§^ *p* ≤ 0.01. **, ^##^, ^§§^ *p* ≤ 0.001.

Next, we examined the impact of the different diets on plasma biochemistry in fed and fasted mice of both genotypes. First, fasted mice had lower glycaemia than fed mice, regardless of the diet and genotype (Figure 2a). In addition, *Pparα^−/−^* mice were hypoglycaemic compared to *Pparα^+/+^* mice after fasting, but they were not different from wild-type when fed ad libitum. However, for mice fed a high PUFAs diet (FISH), hypoglycaemia was not significantly different in fasted *Pparα^−/−^* mice compared to wild-type mice, though there was a trend towards reduced glycaemia. Second, though diet and genotype strongly influenced the levels of free fatty acids in the fed state, fasting strongly elevated them in the plasma, independent of the diet and genotype (Figure 2b). Moreover, circulating levels of triglycerides (Figure 2c), LDL cholesterol (Figure 2d), HDL cholesterol (Figure 2e), and total cholesterol (Figure 2d) were influenced by the genotype, diet, and fasting. Finally, in *Pparα^−/−^* mice, fasting induced elevated plasma aspartate transaminase (AST) and alanine transaminase (ALT) activity, which likely reflects liver damage, as the levels were particularly enhanced in *Pparα^−/−^* mice fed an EFAD or FF diet.

### 2.3. Effect of Dietary Fatty Acids and Fasting on Liver Steatosis

**Figure 3:**
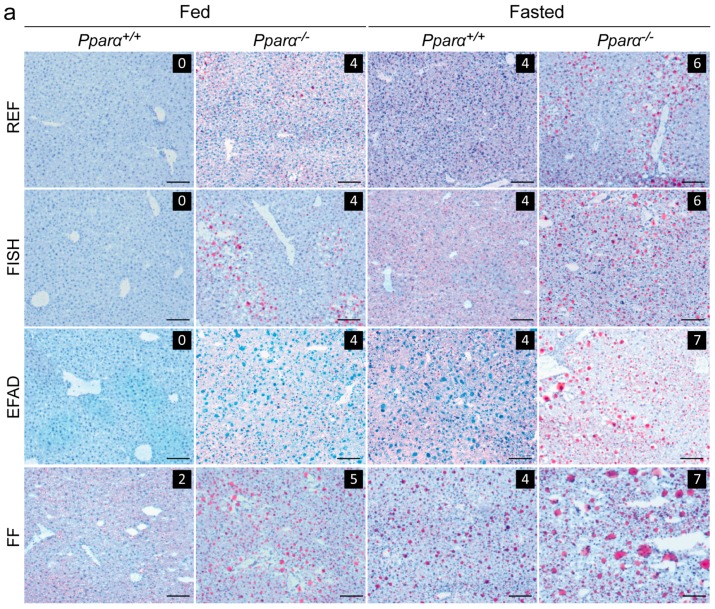

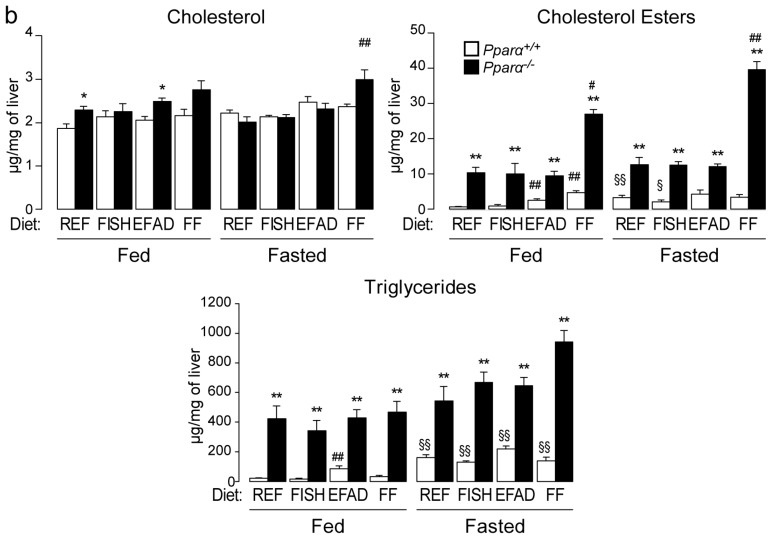
Effect of dietary fat on liver steatosis. Wild-type (*Pparα^+/+^*) and total *Pparα* knockout (*Pparα^−/−^*) mice were fed ad libitum or fasted for 24 h and then euthanized at ZT14. (**a**) Representative pictures of Oil-Red-O stained liver sections. Scale bars = 100 µm. Histological scoring was performed and is noted in the top right of each picture; (**b**) quantification of total cholesterol, cholesterol esters, and triglycerides in the liver by gas chromatography. *n* = 6–8 mice/genotype/diet. Data represent mean ± SEM. * Significant genotype effect, ^#^ significant diet effect, ^§^ significant effect of fasting. *, ^#^, ^§^ *p* ≤ 0.01. **, ^##^, ^§§^ *p* ≤ 0.001. REF: standard diet; FISH: diet enriched in n-3 essential fatty acids from fish oil; EFAD: essential fatty acid-deficient diet; FF: fat-free diet.

We also examined the extent to which hepatic steatosis was influenced by dietary fat and fasting. We performed histological scoring (Figure 3a) and found that, in the fed state, steatosis was only detectable in *Pparα^−/−^* mice, not in wild-type mice, regardless of the diet. In the fasted state, neutral fat accumulation was detected in both wild-type and in *Pparα^−/−^* mice. However, fasting-induced steatosis was more severe in *Pparα^−/−^* mice than in wild-type mice. Hepatic lipid analyses were in agreement with these observations; cholesterol esters and triglycerides were consistently elevated in *Pparα^−/−^* mice compared to wild-type mice in both the fed and fasted state (Figure 3b). In the long term, PPARα deficiency leads to spontaneous steatosis in both fed and fasted mice, regardless of the dietary fat consumed.

### 2.4. Effect of Dietary Fatty Acids and Fasting on Hepatic Gene Expression

**Figure 4:**
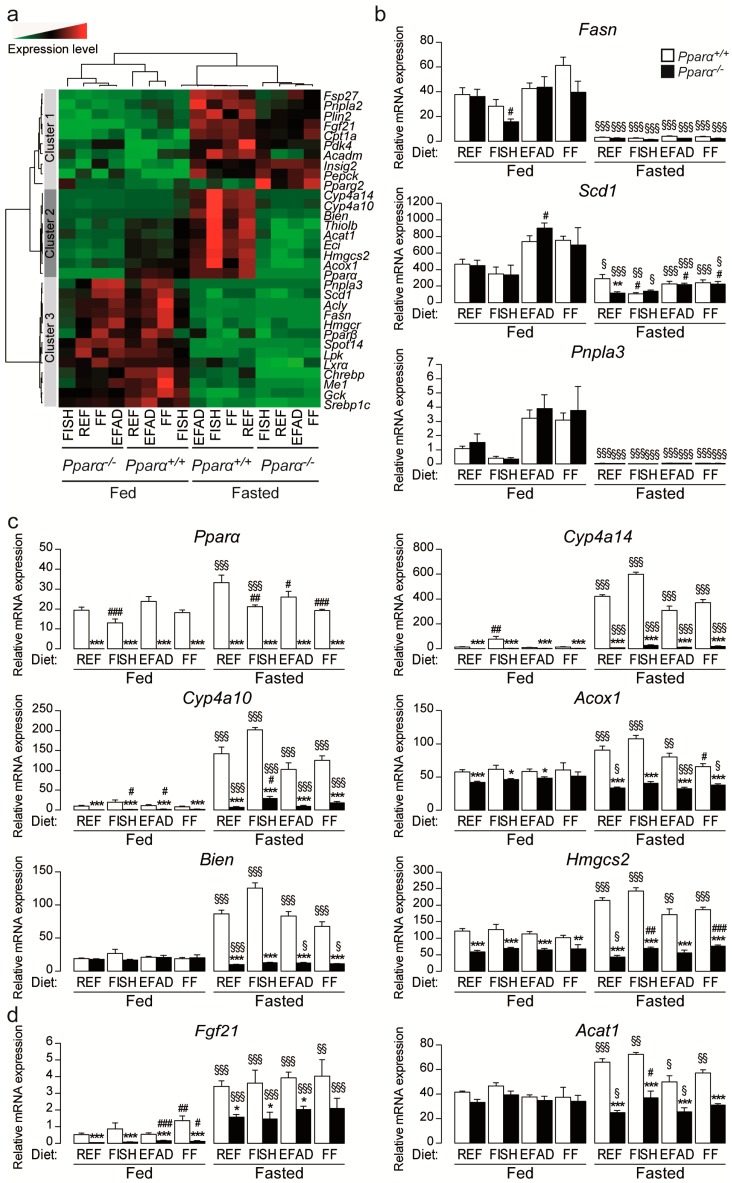
Effect of dietary fat on hepatic gene expression. Wild-type (*Pparα^+/+^*) and total *Pparα* knockout (*Pparα^−/−^*) mice were fed ad libitum or fasted for 24 h and then euthanized at ZT14. (**a**) Hierarchical clustering of hepatic gene expression data determined for 32 genes related to lipid metabolism and nuclear receptor signalling via qRT-PCR of liver samples from male *Pparα^+/+^* and *Pparα^−/−^* mice; (**b**–**d**) Quantification of *Fasn*, *Scd1*, *Pnpla3*, *Pparα*, *Cyp4a14*, *Cyp4a10*, *Acox1*, *Bien*, *Hmgcs2*, *Fgf21*, *Acat1* mRNA expression levels in the liver as determined by qRT-PCR. Data were normalized to the expression of TATA-binding protein (TBP). Data represent mean ± SEM. * significant genotype effect, ^#^ significant effect of diet composition, ^§^ significant effect of fasting. *, ^#^, ^§^ *p* ≤ 0.05. **, ^##^, ^§§^ *p* ≤ 0.01. ***, ^###^, ^§§§^ *p* ≤ 0.001. REF: standard diet; FISH: diet enriched in n-3 essential fatty acids from fish oil; EFAD: essential fatty acid-deficient diet; FF: fat-free diet.

Finally, we investigated whether dietary fat influences gene expression in wild-type and *Pparα^−/−^* mice when fed or fasted. The expression of genes encoding proteins involved in the regulation of the circadian core clock was assessed and showed no difference regardless of diet and genotype (Figure S1a–c). Moreover, the expression of a set of 32 genes involved in hepatic metabolism was measured and presented as a heat map (Figure 4a) to highlight different gene clusters. Genes involved in fatty acid synthesis are grouped in cluster 3. The expression of these lipogenic genes (e.g., *Fasn*, *Scd1*, and *Pnpla3*) was sensitive to dietary fatty acids but did not exhibit dependence on PPARα (Figure 4b). This finding is consistent with their high expression in fed mice and repression during fasting when PPARα is active. Clusters 1 and 2 comprise genes that are up-regulated during fasting (Figure 4a). The expression of PPARα and a number of its target genes involved in fatty acid catabolism was also assessed (Figure 4a, clusters 1 and 2; Figure 4c). Interestingly, the expression of some of the target genes, such as *Cyp4a14*, *Cyp4a10*, *Acox1*, *Bien*, and *Hmgcs2*, was decreased in *Pparα^−/−^* mice compared to wild-type mice. The expression of *Cyp4a14*, a very sensitive PPARα target, was significantly increased in wild-type mice fed the FISH diet, but not in *Pparα^−/−^* mice. The difference in the expression of PPARα target genes between wild-type and *Pparα^−/−^* mice was greater in fasted mice, regardless of diet. Moreover, SIRT1-sensitive genes, such as *Hmgcs2*, *Fgf21*, and *Acat1*, were increased upon starvation regardless of the diet in a PPARα-dependent manner (Figure 4c,d, Figure S1d).

## 3. Discussion

In the liver, fasting triggers strong transcriptional regulation that allows the maintenance of glycaemia and the use of ketone bodies produced through fatty acid catabolism as an alternative energy source. In this process, several transcription factors sense the hormonal and metabolic challenges that occur in food-deprived animals [4]. Hepatocyte PPARα has been shown to play a major role in this adaptation, as PPARα-deficient mice are hypoglycaemic and exhibit impaired fatty acid oxidation that promotes hepatic steatosis during fasting [5,6,14].

Hormones and nutrients, such as fatty acids, have been shown to control PPARα activity in the liver. Glucagon increases hepatic PPARα activity during fasting [15], and several lines of evidence have demonstrated that insulin- and nutrient-sensitive pathways, such as those depending on mTORC1 [16], AKT/PKB [17], and S6 kinase 2 [18], influence the repression of PPARα activity through its co-repressor NCoR1. Sirtuin 1 (SIRT1), a NAD^+^-dependent deacetylase, also controls hepatic fatty acid metabolism by regulating PPARα activity [19]. In addition, PPARα acts as a fatty acid sensor that is sensitive to many species of fatty acids and fatty acid-derived molecules [9,10,11,12,13]. Dietary fatty acids have been shown to influence hepatic PPARα activity, especially fatty acids derived from the n-3 and n-6 series [9,10]. Recently, using hepatocyte-specific deletion of *Pparα*, we showed that adipocyte lipolysis, which is induced in response to a low insulin/glucagon ratio during fasting, produces free fatty acids that activate PPARα in the liver [14]. Furthermore, manipulating hepatic lipase [20,21] and thioesterase [22] activity has been reported to modify hepatocyte PPARα signalling.

PPARα expression has been shown to be circadian [22] and highly inducible in the early nocturnal phase in mice [14]. Therefore, a combination of fatty acid-derived signals and other fatty acid-independent signalling influences PPARα activity during fasting. To the best of our knowledge, whether specific fatty acid series or fatty acid-derived species are highly influential in this response has not been addressed.

Here, we investigated the influence of the hepatic fatty acid profile on liver PPARα activity during fasting. We used different diets with contrasting dietary fatty acids to induce major remodelling of the hepatic fatty acid profile. Once this was achieved, mice were submitted to an acute fasting challenge to test whether specific lipids are required to produce ligands for the PPARα-dependent response in the diurnal phase. Our data show that, on the one hand, the use of FF and EFAD diets long term led to an expected reduction in tissue n-6 and n-3 PUFAs compared to the REF diet as we previously demonstrated [23]. On the other hand, a diet high in n-3 PUFAs increased the relative abundance of the lipid-derived n-3 series in the liver. These changes are of interest because PUFAs, especially those of the n-3 series, are thought to activate PPARα-dependent responses [9]. Our results suggest that hypoglycaemia and steatosis occur in *Pparα^−/−^* mice regardless of the composition of their diet. Interestingly, we also found that the fasting-induced PPARα-mediated transcriptional response occurs independent of the diet, suggesting that the effect of dietary fatty acids on PPARα target gene expression is very modest compared to fasting. Thus, the key signalling pathway that PPARα triggers in response to fasting does not depend on the specific molecular species of fatty acids.

Interestingly, we found that the FF and EFAD diets increase liver damage induced by fasting when PPARα is lacking, as reflected by the plasma levels of ALT and AST. This finding implies that essential fatty acid-derived molecules may be required to induce lipoprotective mechanisms involving PPARα. Hepatic induction of FGF21 by SIRT1 has been shown to protect from liver steatosis [24]. During fasting hepatic SIRT1 regulates glucose and lipid homeostasis [25,26,27]. Glycaemia and the expression of genes such as *Fgf21*, *Hmgcs2*, and *Acat1* are known to be sensitive to SIRT1 during fasting are not modified by the diet. In addition, circadian clock genes such as *Rev-erbα* and *Rev-erbβ*, as well as *Bmal1*, which are sensitive to SIRT1 activity, are not modified either [28,29]. This suggests that hepatic damages induced by essential fatty acid deficiency are not due to a specific effect on SIRT1 activity. However, SIRT1 sensitive pathways seem down-regulated in fasted PPARα^−/−^ mice. This suggests that during starvation SIRT1 is likely to influence hepatic function partly by modulating PPARα activity.

Saturated fatty acids, and possibly medium chain saturated fatty acids, have already been shown to be hepatotoxic [29]. Our work suggests that they may become particularly harmful for the liver if adipocyte lipolysis occurs in the absence of PPARα. PPARα hepatic activity has been shown to be potently inhibited by the stress activated JNK signalling pathway in pre-clinical model of diabetes [30] that results in enhanced adipose tissue lipolysis. Therefore, this work further supports that essential fatty acid intake might be important to prevent deleterious progression of non-alcoholic liver diseases [31], particularly in patients with type II diabetes.

## 4. Materials and Methods

### 4.1. Animals and Diets

In vivo studies were conducted under EU guidelines for the use and care of laboratory animals and approved by an independent ethics committee (Ethics committee of Pharmacology and Toxicology number 86, Toulouse Midi-Pyrénées. Permission date 2014/09/10). *Pparα*-deficient and wild-type mice with a C57Bl6/J genetic background were bred at INRA’s transgenic rodent facility and maintained at 22 ± 2 °C. Eight-week-old male mice (*n* = 6–8) were given experimental diets ad libitum for 10 weeks (pellets prepared by UPAE-INRA, Jouy-en-Josas, France, replaced twice a week) with free access to water. All diets were isocaloric and contained 5% fat (*w*/*w*). Oils used for experimental diet preparations were: grape seed and colza oils (50/50) for the REF diet, hydrogenated coconut oil for the EFAD diet, and grape seed/colza/fish oils (40/40/20) for the FISH diet. The FF diet was devoid of oil. The fish oil was obtained from Polaris (Quimper, France). Diet and oil compositions are given in Tables S2 and S3, respectively. The fatty acid composition was controlled via gas chromatographic analysis of organic extracts of the manufactured food pellets.

### 4.2. Blood and Tissue Samples

Mice were euthanized at Zeitgeber time (ZT) 14, with ZT0 being when the lights are turned on and ZT12 when lights are turned off. Prior to sacrifice, blood was collected from the submandibular vein using a lancet into EDTA-coated tubes (BD Microtainer, K2E tubes, Franklin Lake, NJ, USA). Plasma was prepared by centrifugation (1500× *g*, 10 min, 4 °C) and stored at −80 °C. Following euthanasia by cervical dislocation, the organs were removed, weighed, dissected when necessary, and prepared for histological analysis or snap-frozen in liquid nitrogen and stored at −80 °C.

### 4.3. RNA Extraction and RT-qPCR

Total RNA was extracted using TRIzol reagent (Invitrogen, Carlsbad, CA, USA). For real-time quantitative PCR, 2 µg of RNA was reverse-transcribed using the High-Capacity cDNA Reverse Transcription Kit (Applied Biosystems, Foster City, CA, USA). The SYBR Green assay primers used in the study are presented in Online Table S4. Amplification was performed using an ABI Prism 7300 Real-Time PCR System (Applied Biosystems) using SYBR Green Master Mix (Applied Biosystems) as followed: 10 min at 95 °C, then 40 cycles of 15 s at 95 °C, 60 s at 60 °C, ending by the dissociation curve. All primers were designed with a Tm value of 60 °C and used at 300 mM. The qPCR data were analysed by LinRegPCR.v2015.3 (Heart Failure Research Center, Amsterdam, The Netherlands) and normalized to the levels of TATA-box-binding protein mRNA.

### 4.4. Biochemical Analysis

AST, ALT, total cholesterol, LDL cholesterol, and HDL cholesterol levels were determined in plasma using a COBAS-MIRA+ biochemical analyser (Anexplo facility).

### 4.5. Histology

Snap-frozen liver samples in isopentane pre-cooled in liquid nitrogen were embedded in Tissue Tek OCT compound (Sockura Finetek, Tokyo, Japan). Sections (5 µm, Leica RM2145 microtome, Wetzlar, Germany) were stained with Oil-Red-O and counterstained with haematoxylin before visualization with a Leica DFC300 camera (Leica). Histological scoring was established according to Kleiner et al. [32].

### 4.6. Liver Neutral Lipids Analysis

Tissue samples were homogenized in methanol with 5 mM EGTA (2:1, *v*/*v*) and the lipids (corresponding to an equivalent of 2 mg tissue) extracted following the Bligh–Dyer method using chloroform, methanol, and water (2.5:2.5:2.1, *v*/*v*/*v*) in the presence of internal standards glyceryl trinonadecanoate, stigmasterol, and cholesteryl heptadecanoate (Sigma, Saint Louis, MO, USA). Triglycerides, free cholesterol, and cholesterol esters were analysed by gas-liquid chromatography using a Focus Thermo Electron system with a Zebron-1 Phenomenex fused-silica capillary column (5 m, 0.32 mm i.d., 0.50 mm film thickness). The oven temperature was programmed to increase from 200 to 350 °C at 5 °C/min. The carrier gas was hydrogen (0.5 bar). The injector and detector temperatures were 315 and 345 °C, respectively.

### 4.7. Liver Fatty Acid Analysis

To measure total hepatic fatty acid methyl ester (FAME) molecular species, lipids corresponding to an equivalent of 1 mg of liver were extracted in the presence of glyceryl triheptadecanoate (0.5 μg) as an internal standard. The lipid extract was transmethylated with 1 mL of BF3 in methanol (14% solution; Sigma) and 1 mL of hexane for 60 min at 100 °C and evaporated to dryness. The FAMEs were extracted with hexane and water (2:1). The organic phase was evaporated to dryness and dissolved in 50 µL ethyl acetate. A sample (1 µL) of total FAME was analysed by gas-liquid chromatography (Clarus 600 Perkin Elmer system using Famewax RESTEK fused silica capillary columns (30 m × 0.32 mm i.d., 0.25 µm film thickness)). The oven temperature was programmed from 110 to 220 °C at a rate of 2 °C per minute. The carrier gas was hydrogen (0.5 bar). The injector and detector were at 225 and 245 °C, respectively.

### 4.8. Statistical Analysis

Data were analysed using R (http://www.r-project.org). Differential effects were assessed on log2-transformed data using analysis of variance followed by Student’s *t*-tests with a pooled variance estimate. The *p-*values from *t*-tests were adjusted by Benjamini-Hochberg correction. *p* ≤ 0.05 was considered significant. Hierarchical clustering of gene expression and hepatic lipid quantification were established with R packages Geneplotter and Marray (https://www.bioconductor.org/). Ward’s algorithm modified by Murtagh and Legendre was used for clustering. All of the data represented on the heat maps have a *p* ≤ 0.05 for one or more comparisons by analysis of variance.

## 5. Conclusions

Essential fatty acid (n-6 and n-3 series) intake is important to prevent deleterious progression of non-alcoholic liver diseases. PPARα activity is regulated by fatty acids and protects from steatosis, suggesting that dietary essential fatty acids may control the activity of this nuclear receptor. However, the potent signalling involved in triggering PPARα activity during fasting remains inducible in mice with essential fatty acid deficiency. Therefore, PPARα ligands are unlikely to be exclusively derived from dietary PUFAs.

## Supplementary Materials

Supplementary materials can be found at www.mdpi.com/1422-0067/17/10/1624/s1.
