# Supplementary material for: Hepatic Fasting-Induced PPARα Activity Does Not Depend on Essential Fatty Acids
Source: Int J Mol Sci. 2016 Sep 24;17(10):1624. doi: 10.3390/ijms17101624 (PMC5085657; doi:10.3390/ijms17101624)
Supplement: Supplementary file 1 [file ijms-17-01624-s001.pdf]

Supplementary Materials: Hepatic Fasting-Induced PPARα Activity Does Not Depend on Essential Fatty Acids

Arnaud Polizzi, Edwin Fouché, Simon Ducheix, Frédéric Lasserre, Alice P. Marmugi, Laila Mselli-Lakhal, Nicolas Loiseau, Walter Wahli, Hervé Guillou and Alexandra Montagner

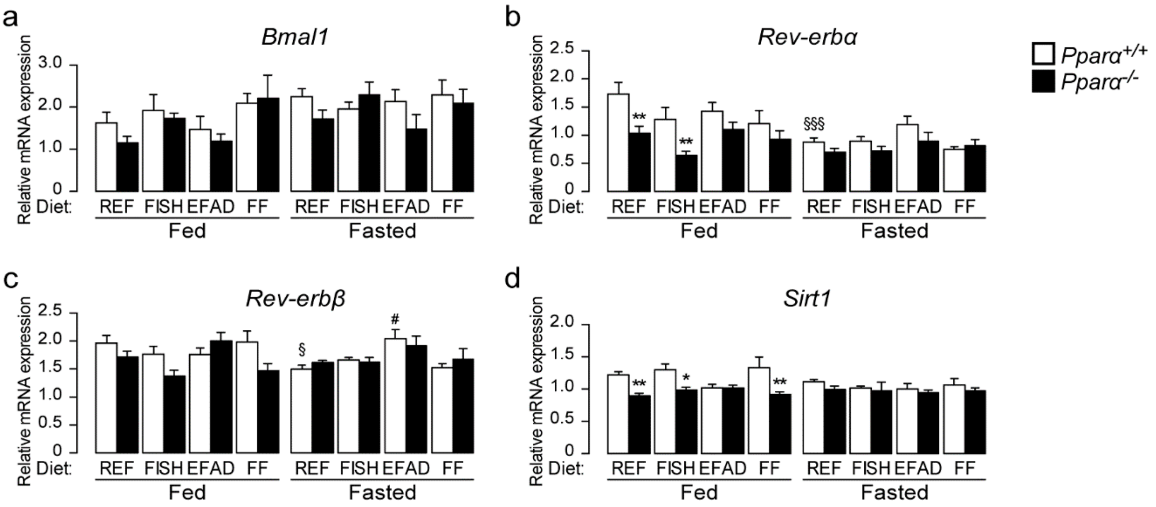

Figure S1. Effect of dietary fat on hepatic gene expression. Wild-type (*Pparα*$^{+/+}$) and total *Pparα* knockout (*Pparα*$^{-/-}$) mice were fed ad libitum or fasted for 24 h and then euthanized at ZT14. Quantification of *Bmal1* (**a**); *Rev-erbα* (**b**); *Rev-erbβ* (**c**); and *Sirt1* (**d**) mRNA expression levels in the liver as determined by qRT-PCR. Data were normalized to the expression of TATA-binding protein (TBP). Data represent mean ± SEM. * significant genotype effect, # significant effect of diet composition, § significant effect of fasting. *, #, § $p \leq 0.05$. ** $p \leq 0.01$. §§§ $p \leq 0.001$. REF: standard diet; FISH: diet enriched in n-3 essential fatty acids from fish oil; EFAD: essential fatty acid-deficient diet; FF: fat-free diet.

Table S1. Liver composition in FA from liver of wild-type (*Ppar*α$^{+/+}$) and total *Ppar*α knockout (*Ppar*α$^{-/-}$) mice were fed ad libitum or fasted for 24 h and killed at ZT14. Transmethylated fatty acids from liver lipid extract were quantified by gas chromatography. Data shown as mean ± SEM. * significant genotype effect, $^{\#}$ significant effect of diet composition, § significant effect of fasting. *, $^{\#}$, § $p \leq 0.01$, **, $^{\#\#}$, §§ $p \leq 0.001$. REF: standard diet; FISH: diet enriched in n-3 essential fatty acids from fish oil; EFAD: essential fatty acids deficient diet; FF: fat free diet.

	**Fed**							
	**REF**		**FISH**		**EFAD**		**FF**	
	***Ppar*α$^{+/+}$**	***Ppar*α$^{-/-}$**	***Ppar*α$^{+/+}$**	***Ppar*α$^{-/-}$**	***Ppar*α$^{+/+}$**	***Ppar*α$^{-/-}$**	***Ppar*α$^{+/+}$**	***Ppar*α$^{-/-}$**
C14:0	0.19 ± 0.044	0.51 ± 0.029 **	0.02 ± 0.008 $^{\#\#}$	0.35 ± 0.082 **	0.61 ± 0.11 $^{\#\#}$	1.99 ± 0.07 **,$^{\#\#}$	0.12 ± 0.022	0.61 ± 0.039 **
C16:0	19.9 ± 0.99	21.8 ± 0.85	18.4 ± 1.01	24.9 ± 1.76 **	20.9 ± 0.56	23.1 ± 0.72	18 ± 0.6	23.1 ± 0.86 **
C16:1 n-9	0.85 ± 0.049	1.19 ± 0.121	0.99 ± 0.069	1.09 ± 0.147	0.93 ± 0.156	1.28 ± 0.069	1.36 ± 0.082	1.56 ± 0.205
C16:1 n-7	2.1 ± 0.18	4.5 ± 0.32 **	1.8 ± 0.43	3.8 ± 0.73 **	4.6 ± 0.31 $^{\#\#}$	10.5 ± 0.48 **,$^{\#\#}$	4.1 ± 0.23 $^{\#\#}$	8.7 ± 0.78 **,$^{\#\#}$
C18:0	10.2 ± 0.43	4.4 ± 0.57 **	10.7 ± 0.98	8.3 ± 1.84	7.3 ± 0.68	2.7 ± 0.3 **	7.3 ± 0.64	5.5 ± 1.61
C18:1 n-9	23.2 ± 1.27	34.9 ± 0.9 **	18.9 ± 2.38	26.4 ± 3.51 *,$^{\#\#}$	34.5 ± 1.71 $^{\#\#}$	38.1 ± 0.76	38.9 ± 1.58 $^{\#\#}$	38.7 ± 1.47
C18:1 n-7	4.6 ± 0.29	6.4 ± 0.31 *	2.8 ± 0.34 $^{\#\#}$	3.7 ± 0.45 $^{\#\#}$	10.3 ± 0.43 $^{\#\#}$	14.8 ± 0.52 **,$^{\#\#}$	11.5 ± 0.26 $^{\#\#}$	16.5 ± 0.88 *,$^{\#\#}$
C18:2 n-6	13 ± 0.34	17.4 ± 1.07	11.3 ± 0.8	15.4 ± 1.09	3.5 ± 0.37 $^{\#\#}$	2.4 ± 0.26 $^{\#\#}$	2.1 ± 0.3 $^{\#\#}$	2 ± 0.3 $^{\#\#}$
C18:3 n-6	0.26 ± 0.014	0.15 ± 0.027 *	0.1 ± 0.018 $^{\#\#}$	0.06 ± 0.004 $^{\#\#}$	0.07 ± 0.01 $^{\#\#}$	0.03 ± 0.006 **,$^{\#\#}$	0.04 ± 0.006 $^{\#\#}$	0.02 ± 0.004 $^{\#\#}$
C18:3 n-3	0.21 ± 0.021	0.67 ± 0.042 **	0.33 ± 0.017 $^{\#}$	0.95 ± 0.104 **	0.08 ± 0.017 $^{\#\#}$	0.13 ± 0.01 **,$^{\#\#}$	0.2 ± 0.03	0.11 ± 0.011 *,$^{\#\#}$
C20:0	0.74 ± 0.171	0.81 ± 0.161	0.26 ± 0.054	0.28 ± 0.027	0.81 ± 0.184	0.77 ± 0.174	0.06 ± 0.009 $^{\#}$	0.02 ± 0.002 $^{\#\#}$
C20:1 n-9	0.41 ± 0.084	0.55 ± 0.058 *	0.55 ± 0.034	0.71 ± 0.055	0.93 ± 0.068 $^{\#\#}$	0.92 ± 0.088 $^{\#}$	1.41 ± 0.1 $^{\#\#}$	0.78 ± 0.085 *
C20:2 n-6	0.47 ± 0.048	0.32 ± 0.042	0.34 ± 0.03	0.21 ± 0.035	2.88 ± 0.424 $^{\#\#}$	0.41 ± 0.074 **	4.34 ± 0.415 $^{\#\#}$	0.23 ± 0.041 **
C20:3 n-9	0.0081 ± 0.0017	0.0094 ± 0.0024	0.0046 ± 0.0016	0.0012 ± 0.0005	0.11 ± 0.016 $^{\#\#}$	0.032 ± 0.0064 $^{\#}$	0.1506 ± 0.0126 $^{\#\#}$	0.0097 ± 0.0016 *
C20:3 n-6	1.68 ± 0.081	0.41 ± 0.064 **	1.46 ± 0.119	0.33 ± 0.109 **	1.09 ± 0.144 $^{\#}$	0.19 ± 0.031 **,$^{\#\#}$	0.66 ± 0.104 $^{\#\#}$	0.13 ± 0.02 **,$^{\#\#}$
C20:4 n-6	13.9 ± 0.71	3.2 ± 0.65 **	6.3 ± 0.8 $^{\#\#}$	1.7 ± 0.99 **,$^{\#\#}$	6.9 ± 0.77 $^{\#\#}$	1.4 ± 0.2 **,$^{\#\#}$	5.6 ± 0.64 $^{\#\#}$	1 ± 0.2 **,$^{\#\#}$
C20:5 n-3	0.1 ± 0.011	0.09 ± 0.014	4.49 ± 0.509 $^{\#\#}$	2.47 ± 0.603 $^{\#\#}$	0.03 ± 0.007 $^{\#\#}$	0.01 ± 0.003 $^{\#\#}$	0.01 ± 0.006 $^{\#\#}$	0.01 ± 0.003 $^{\#\#}$
C22:4 n-6	0.48 ± 0.063	0.18 ± 0.04 **	0.09 ± 0.015 $^{\#\#}$	0.07 ± 0.014 $^{\#}$	0.15 ± 0.028 $^{\#\#}$	0.03 ± 0.005 **,$^{\#\#}$	0.09 ± 0.016 $^{\#\#}$	0.02 ± 0.006 **,$^{\#\#}$
C22:5 n-6	0.76 ± 0.195	0.13 ± 0.031 **	0.08 ± 0.016 $^{\#\#}$	0.05 ± 0.007 $^{\#}$	0.85 ± 0.178	0.19 ± 0.019 **	0.87 ± 0.07	0.12 ± 0.028 **
C22:5 n-3	0.23 ± 0.013	0.12 ± 0.021 *	1.92 ± 0.082 $^{\#\#}$	1.94 ± 0.311 $^{\#\#}$	0.04 ± 0.008 $^{\#\#}$	0.01 ± 0.002 **,$^{\#\#}$	0.01 ± 0.006 $^{\#\#}$	0 ± 0.001 $^{\#\#}$
C22:6 n-3	6.3 ± 0.41	1.7 ± 0.37 **	18.7 ± 1.75 $^{\#\#}$	6.9 ± 2.28 **,$^{\#\#}$	2.9 ± 0.3 $^{\#\#}$	0.6 ± 0.09 **,$^{\#\#}$	2.7 ± 0.32 $^{\#\#}$	0.4 ± 0.09 **,$^{\#\#}$

Table S2. *Cont.*

	Fasted							
	REF		FISH		EFAD		FF	
	Pparα$^{+/+}$	***Pparα***$^{-/-}$	***Pparα***$^{+/+}$	***Pparα***$^{-/-}$	***Pparα***$^{+/+}$	***Pparα***$^{-/-}$	***Pparα***$^{+/+}$	***Pparα***$^{-/-}$
C14:0	0.35 ± 0.043 §§	0.53 ± 0.077	0.36 ± 0.033 §§	0.55 ± 0.027	1.3 ± 0.084 ##,§	1.96 ± 0.126 ##	0.47 ± 0.052 §§	0.83 ± 0.037
C16:0	20.8 ± 0.62	18 ± 0.78 §	22.7 ± 0.57 §	25.4 ± 1.15 ##	20.7 ± 0.43	19.9 ± 0.21	20.4 ± 0.29	20.3 ± 1.1
C16:1 n-9	1.14 ± 0.138	1.32 ± 0.113	1.13 ± 0.068	1.13 ± 0.132	1.26 ± 0.063	1.1 ± 0.115	1.53 ± 0.121	1.74 ± 0.12
C16:1 n-7	4.6 ± 0.34 §§	7.2 ± 0.52 *,§	4.4 ± 0.08 §§	4.6 ± 0.28 #	11.7 ± 0.44 ##,§§	17.4 ± 1.02 *,##,§§	8.3 ± 0.68 ##,§§	11.6 ± 1.68 #
C18:0	3.8 ± 0.3 §§	3.5 ± 0.57	3.9 ± 0.21 §	5.7 ± 1.27	3.4 ± 0.17 §	2.9 ± 0.65	4.4 ± 0.81	5.7 ± 2.38
C18:1 n-9	34.6 ± 1.46 §§	36.5 ± 0.71	23.9 ± 0.87 ##	29.4 ± 2.02	40.5 ± 0.52	38.8 ± 0.72	41.3 ± 1.23	40.5 ± 1.66
C18:1 n-7	3.4 ± 0.22 §	4.9 ± 0.4 *	1.9 ± 0.25 ##,§	3.1 ± 0.36 **,##	6.1 ± 0.48 ##,§§	11.5 ± 0.98 **,##	6.5 ± 0.36 ##,§§	14.5 ± 0.54 **,##
C18:2 n-6	18 ± 1.11	21.8 ± 1.08	18.9 ± 0.73 §	16.3 ± 0.91	3.8 ± 0.27 ##	2.8 ± 0.22 ##	3.8 ± 0.3 ##,§	2.4 ± 0.72 *,##
C18:3 n-6	0.78 ± 0.125 §§	0.46 ± 0.05 §§	0.28 ± 0.046 ##,§§	0.1 ± 0.017 **,##	0.39 ± 0.046 ##,§§	0.18 ± 0.02 **,##,§§	0.31 ± 0.037 ##,§§	0.12 ± 0.037 **,##,§§
C18:3 n-3	0.55 ± 0.038 §§	1.02 ± 0.064 **,§	0.88 ± 0.056#,§§	1.14 ± 0.067	0.07 ± 0.007 ##	0.11 ± 0.013*,##	0.09 ± 0.007 ##,§§	0.12 ± 0.007##
C20:0	0.37 ± 0.1	0.47 ± 0.115	0.05 ± 0.004	0.35 ± 0.066	0.49 ± 0.151	0.42 ± 0.107	0.02 ± 0.005 #	0.01 ± 0.003 #
C20:1 n-9	0.25 ± 0.028	0.37 ± 0.039	0.31 ± 0.034 §	0.67 ± 0.052 **,#	0.32 ± 0.014 §§	0.62 ± 0.038 **,#	0.45 ± 0.072 #,§§	0.59 ± 0.083
C20:2 n-6	0.24 ± 0.01 §§	0.22 ± 0.017	0.12 ± 0.012 #,§§	0.15 ± 0.014	1.95 ± 0.176 ##	0.27 ± 0.02 **	2.16 ± 0.338 ##,§	0.16 ± 0.011 **
C20:3 n-9	0.002 ± 0.0003	0.0039 ± 0.0009	0.0021 ± 0.0005	0.001 ± 0.0006	0.0576 ± 0.0153 ##	0.0129 ± 0.0019	0.0508 ± 0.0192 ##	0.0042 ± 0.0007
C20:3 n-6	0.39 ± 0.021 §§	0.17 ± 0.012 **,§§	0.41 ± 0.042 §§	0.18 ± 0.016 **	0.42 ± 0.033 §§	0.06 ± 0.004 **,##,§§	0.38 ± 0.069 §	0.03 ± 0.004 **,##,§§
C20:4 n-6	5.7 ± 0.41 §§	1.6 ± 0.14 **	2.6 ± 0.14 ##,§	0.7 ± 0.05 **,##	4 ± 0.14	0.9 ± 0.05 **,#	5.6 ± 0.77	0.6 ± 0.11 **,##
C20:5 n-3	0.15 ± 0.012	0.08 ± 0.007	3.46 ± 0.152 ##	2.27 ± 0.366 ##	0.02 ± 0.002 ##	0.01 ± 0.001 ##	0.02 ± 0.002 ##	0 ± 0.001 *,##
C22:4 n-6	0.19 ± 0.014 §	0.13 ± 0.018	0.04 ± 0.004 ##	0.05 ± 0.007	0.14 ± 0.036	0.02 ± 0.004 **,##	0.08 ± 0.011 #	0.01 ± 0.005 **,##
C22:5 n-6	0.2 ± 0.02 §§	0.08 ± 0.018 **	0.06 ± 0.006 ##	0.05 ± 0.006	0.73 ± 0.146 ##	0.1 ± 0.006 **	0.59 ± 0.104 ##	0.07 ± 0.007 **
C22:5 n-3	0.26 ± 0.025	0.11 ± 0.012 *	1.65 ± 0.094 ##	1.94 ± 0.332 ##	0.09 ± 0.013 ##,§	0.01 ± 0.001 **,##	0.07 ± 0.001 ##,§§	0.01 ± 0.003 **,##
C22:6 n-3	3.8 ± 0.26	1 ± 0.09 **	12.5 ± 0.7 ##	5.7 ± 0.89 **,##	2.1 ± 0.09 #	0.3 ± 0.02 **,##	3.1 ± 0.39	0.2 ± 0.03 **,##

Table S2. Diet compositions.

% (*w*/*w*)	Fat Free Diet (FF)	CTRL, FISH, EFAD Diets
Cellulose	2	2
Casein	22	22
Starch	46.2	43.5
Methionin	0.2	0.2
Sucrose	23.1	21.8
Minerals	4.5	4.5
Vitamins	1	1
Oil	0	5
Agar-Agar	1	0

Table S3. Relative fatty acid composition of dietary oils.

Diet	REF	EFAD	FISH
12:00	0	47.7	0
14:00	0	23	0
16:00	5.9	13.1	4.4
18:00	2.8	15.3	2.1
18:1w9	39.7	0.9	30.5
18:2w6	47.0	0	34.7
18:3w3	4.6	0	3.4
20:5w3	0	0	5.3
22:6w3	0	0	19.5

Table S4. Oligonucleotide sequences for the real time PCR. Oligonucleotides (Sigma Aldrich, St. Louis, MO, USA) were designed with Primer Express 2.0 software (Applied Biosystems, Foster City, CA, USA).

Gene	NCBI Refseq	Forward Primer (5′–3′)	Reverse Primer (5′–3′)
Acly	NM_134037	AAAGCTTGGCCTCGTCGG	GGGACGAAGGGTTCAATGAGA
Acox1	NM_015729	CAGACCCTGAAGAAATCATGTGG	CAGGAACATGCCCAAGTGAAG
Alas1	NM_020559	CAAAGAAACCCCTCCAGCC	GCTGTGTGCCGTCTGGAGT
Bien	NM_023737	CGTCTCCTCGGTTGGTGTTC	ATTATCTTCTTTGCAGTATCTAGCTGCTT
Bmal1	NM_007489	CAAACTACAAGCCAACATTTCTATCAG	TCGGTCACATCCTACGACAAAC
Cd36	NM_007643	GTTAAACAAAGAGGTCCTTACACATACAG	CAGTGAAGGCTCAAAGATGGC
Chrebp	NM_021455	ACTCAGGGAATACACGCCTACAG	GAAGAAGGAATTCAGAGCTCAGAAA
Cpt1a	NM_013495	GAAGAAGAAGTTCATCCGATTCAAG	GATATCACACCCACCACCACG
Cyp4a10	NM_010011	TCCAGCAGTTCCCATCACCT	TTGCTTCCCCAGAACCATCT
Cyp4a14	NM_007822	TCAGTCTATTTCTGGTGCTGTTC	GAGCTCCTTGTCCTTCAGATGGT
Eci	NM_010023	GTTCACCATCAGCCTGGAGAAG	AGAAGATACCCGGGCATTCC
Fasn	NM_007988	AGTCAGCTATGAAGCAATTGTGGA	CACCCAGACGCCAGTGTTC
Fgf21	NM_020013	AAAGCCTCTAGGTTTCTTTGCCA	CCTCAGGATCAAAGTGAGGCG
Fsp27	NM_178373	CATGAAGTCTCTCAGCCTCCTGTA	CAGCTGTTGGGTCACCACTG
Hmgcr	NM_008255	CTTGTGGAATGCCTTGTGATTG	GAAGAATGTCATGAACACAAAGTAGTTG
Hmgcs2	NM_008256	TGCAGGAAACTTCGCTCACA	AAATAGACCTCCAGGGCAAGGA
Insig2	NM_133748	TGTATATTTTTTGCTGGAGGCATAAC	TTCAGCAATAACTTTGCATTCATACAT
Lpk	NM_013631	TCGACTCAGAGCCTGTGGC	AGTCGTGCAATGTTCATCCCT
Lxra	NM_013839	GGAGTGTCGACTTCGCAAATG	TCAAGCGGATCTGTTCTTCTGAC
Lxrb	NM_009473	ATGCGGGAGCAGTGCG	CCGGCCTGAGCTGCTG
Me1	NM_008615	CATTCGAGGCGTTTCGTTG	CAGGTAGGATCTGGTCATAATTAGTGC
Pdk4	NM_013743	ATCGCCAGAATTAAACCTCACAC	TGGATTGGTTGGCCTGGA
Pepck	NM_011044	GAACCCCAGCCTGCCC	GAGCAACTCCAAAAAACCCG
Plin2	NM_007408	CCATTTCTCAGCTCCACTCCAC	GTGTCGTCGTAGCCGATGC
Pnpla2	NM_001163689	AGTGTCCTTCACCATCCGCTT	GGATATCTTCAGGGACATCAGGC
Pnpla3	NM_054088	ACGCGGTCACCTTCGTGT	AGCCCGTCTCTGATGCACTT
Ppara	NM_011144	CCCTGTTTGTGGCTGCTATAATTT	GGGAAGAGGAAGGTGTCATCTG
Pparb	NM_011145	AAGTGGCCATGGGTGACG	TGGTCCAGCAGGGAGGAAG
Pparg2	NM_011146	ATGGGTGAAACTCTGGGAGATTCT	CTTGGAGCTTCAGGTCATATTTGTA
Reverba	NM_145434	CAGCTGGTGAAGACATGACGAC	GGAGGAGCCACTAGAGCCAA
Reverbb	NM_011584	CGCCATGGAGCTGAACG	GACAAGAGGCAGGGCTGGA
Scd1	NM_009127	CAGTGCCGCGCATCTCTAT	CTGACTGGCAAATATAGCTGTATCCT
Spot14	NM_009381	AACGGAGGAGGCCGAAGAAG	GTTGATGCACCTCGGGGTCT
Tbp	NM_013684	ACTTCGTGCAAGAAATGCTGAA	GCAGTTGTCCGTGGCTCTCT
Srebp1c	NM_011480	GGAGCCATGGATTGCACATT	GCTTCCAGAGAGGAGGCCAG
Thiolb	NM_146230	ACATCTCCGTGGGCAATGTG	TGTCTGTTGACCGTAGACAAAGGT
